# Quality Evaluation of Randomized Controlled Trials of *Rhodiola* Species: A Systematic Review

**DOI:** 10.1155/2021/9989546

**Published:** 2021-07-01

**Authors:** Xiuzhu Li, Weijie Chen, Yingqi Xu, Zuanji Liang, Hao Hu, Shengpeng Wang, Yitao Wang

**Affiliations:** ^1^Institute of Chinese Medical Sciences, State Key Laboratory of Quality Research in Chinese Medicine, University of Macau, Macao, China; ^2^Department of Pharmacy, Faculty of Science, National University of Singapore, Singapore

## Abstract

**Background:**

*Rhodiola* is a worldwide used medicinal plant for its various medicinal functions, and the number of randomized controlled trials (RCTs) of *Rhodiola* is increasing in recent years. This study aims to evaluate the reporting quality and risk of bias of the current RCT reports of different *Rhodiola* species.

**Methods:**

Six databases including Embase, PubMed, Web of Science, the Cochrane Library, ClinicalTrial.gov, and China National Knowledge Infrastructure were searched to identify RCTs that used *Rhodiola* as a single intervention and were published in English or Chinese from inception to December 2020. The Consolidated Standards of Reporting Trials (CONSORT) 2010 statement was used as the checklist for assessment, and a scoring system was applied to the evaluation of RCTs. Score 0 represents no reporting or inadequate reporting, and score 1 represents adequate reporting. The risk of bias of the included studies was also assessed using the Cochrane Risk of Bias tool.

**Results:**

A total of 39 RCTs were included in this study, including 23 RCTs of *Rhodiola rosea* (*R*. *rosea*), 8 RCTs of *Rhodiola crenulata* (*R*. *crenulata*), and 8 RCTs of *Rhodiola wallichiana* (*R*. *wallichiana*). None of the included studies met all the CONSORT statement criteria, and the reporting quality of RCTs of the three *Rhodiola* species was all generally poor. Based on the risk of bias assessment, the majority of included studies were judged to have an unclear risk of bias in most domains due to inadequate reporting.

**Conclusions:**

There is inadequate reporting among the included RCTs of different *Rhodiola* species, and RCTs of *Rhodiola* with higher reporting quality and better methodological quality are needed.

## 1. Introduction

The *Rhodiola* genus, belonging to the Crassulaceae family, is a medicinal plant that has been traditionally used as an adaptogen and tonics, as well as in remedies of anti-inflammatory and antidepression in Europe and Asia since ancient times [1, 2]. *Rhodiola* is inclined to grow in mountainous areas of low temperature such as precipices, tundra, riverbanks, and rock ledges in the northern hemisphere including Asia, North and Central Europe, and North America [3]. It consists of more than 100 species, of which about 20 species are used as traditional medicines, including *Rhodiola rosea* (*R*. *rosea*), *Rhodiola crenulata* (*R*. *crenulata*), *Rhodiola sacra* (*R*. *sacra*), and *Rhodiola kirilovii* [4, 5]. Growing studies have demonstrated that *Rhodiola* possesses varieties of bioactivities such as antistress, antifatigue, antioxidant, antitumor, anti‐inflammation, antiaging, antiradiation, and immunomodulatory [2, 6, 7]. Currently, apart from the traditional applications, *Rhodiola* is also used to treat bronchial asthma and coronary heart disease, to improve chronic fatigue syndrome and physical activity, and to alleviate mountain sickness syndrome in clinical practices [8, 9]. Owing to its numerous functions and economic value, *Rhodiola* has been developed into diverse products including drugs, food supplements, food additives, drinks, and cosmetics [10, 11].

In order to evaluate the claimed functions of *Rhodiola*, a randomized controlled trial (RCT) design has been conducted since the 1960s [9]. As the “gold standard” for evaluating the efficacy of most interventions [12], RCTs are increasingly being used in traditional medicine. RCTs with high reporting quality are essential for the interpretation and reproducibility of a trial and for proper healthcare decisions [13]. On the contrary, low-quality RCTs reports may lead to distorted findings of a study, thus drawing misleading conclusions [14]. Besides, it has been previously shown that RCTs of low quality may be included in the meta-analysis, thereby biasing downstream treatment [15]. Therefore, with the necessity to evaluate the quality of RCTs, several quality assessment tools have been developed. The Consolidated Standards of Reporting Trials (CONSORT) statement [16], which was first published in 1996 and updated in 2001 and 2010, is regarded as the “gold standard” for evaluating the reporting quality of RCTs, aiming to help improve the reporting quality of RCTs [17, 18]. Another special assessment tool for methodological quality assessment, namely, the Cochrane Collaboration's Risk of Bias, has been developed to evaluate the study validity in clinical trials by the Cochrane Collaboration since 2008 [19].

In terms of the quality of *Rhodiola* RCTs, there have been several previous studies evaluating the quality of RCTs of *R*. *rosea* [20, 21]. However, as a globally used herbal medicine, different *Rhodiola* species are widely used, and there has been an increasing number of RCTs of *Rhodiola* species in recent years. Furthermore, there has been an increasing demand for *Rhodiola* products due to their multiple medicinal functions, while the similarities and differences between different *Rhodiola* species still need further investigation. There has been little systematic effort so far to evaluate the quality of RCTs of different *Rhodiola* species. Therefore, this study is designed to evaluate the reporting quality and methodological quality of the current RCTs of different *Rhodiola* species by using the CONSORT 2010 statement and the Cochrane Risk of Bias tool, aiming to help improve the quality of future clinical trials of *Rhodiola* species and provide useful information for the utilization and product development of *Rhodiola* species.

## 2. Methods

This study complied with the Preferred Reporting Items for Systematic Reviews and Meta-Analyses (PRISMA) statement [22]. The reporting quality of included RCTs was assessed by utilizing the CONSORT 2010 checklist [16], and the methodological quality was evaluated in accordance with the Cochrane Risk of Bias tool [23].

### 2.1. Eligibility Criteria

Clinical trials with RCT design that used *Rhodiola* as intervention and were published from inception to December 2020 were included for the eligibility screening. Studies were excluded if they met the following criteria: (i) study subjects being not human, (ii) *Rhodiola* combined with other medicines as therapy intervention, (iii) protocol, (iv) not published in English or Chinese, (v) not peer-reviewed journal articles (e.g., theses or dissertations, conference abstracts), (vi) no full-text available, and (vii) no results posted.

### 2.2. Information Sources and Database Search

To identify the eligible studies, literature search was conducted in 6 electronic databases including Embase, PubMed, Web of Science, the Cochrane Library, ClinicalTrial.gov, and China National Knowledge Infrastructure (CNKI). Besides, the references of the final included studies and relevant reviews were also screened for additional eligible studies. Different strategies were used to search the six databases. For the Cochrane Library, “Rhodiola” was used as the search item, and the study type was limited to trials. For the ClinicalTrial.gov, “Rhodiola” was used as the search item in the search bar “Other terms”. The strategies used for the other four databases are presented as follows:

  Embase: (“rhodiola”/exp OR rhodiola) AND (random^*∗*^: ab, ti OR ((clinical NEXT/1 trial^*∗*^): de,ab,ti) OR ′health care quality'/exp)
  PubMed: (Rhodiola) AND ((randomized controlled trial[pt] OR controlled clinical trial[pt] OR randomized[tiab] OR placebo[tiab] OR drug therapy[sh] OR randomly[tiab] OR trial[tiab] OR groups[tiab] NOT (animals [mh] NOT humans [mh])))
  Web of Science: TS = (Rhodiola AND (randomized OR randomized OR randomization OR randomization OR placebo^*∗*^ OR (random^*∗*^ AND (allocat^*∗*^ OR assign^*∗*^)) OR (blind^*∗*^ AND (single OR double OR treble OR triple))))
  CNKI (The search terms are in Chinese): SU = hóng jǐng tiān AND SU = lín chuáng AND FT = suí jī

### 2.3. Study Selection

All the search results were retrieved from the six databases for eligibility screening. After duplicates were removed, the first round of screening was conducted with the title and abstract of each study based on the eligibility criteria mentioned above. In the second round of screening, the full text of the rest of the studies in the first round was further accessed for eligibility. At last, the included studies were grouped into different *Rhodiola* species. The study selection was conducted by two authors (X. L. and W. C.) independently, and any discrepancies were discussed by S. W., X. L., and W. C. to achieve consensus.

### 2.4. Data Items and Extraction

The information of several descriptive characteristics, namely, nonmarketed/marketed products, formulation, focused functions, publication year, locations of RCTs, sample size, and trial length, was extracted from the full text and recorded in a standard form using Microsoft Excel 16.39. This process was performed by two authors (X. L. and W. C.) individually. Any discrepancies were discussed to resolve by S. W., X. L., and W.C. in order to reach an agreement.

### 2.5. CONSORT Items and Scoring System

All the items (37 subdivided items) of the CONSORT statement were included in the reporting quality assessment of RCT reports. To measure the adherence of each study to each item, a scoring system with two grades was used. The reviewers can grade the study for each item with a score 0 or 1. Score 1 indicates that the study adequately reported the item, while score 0 means that the study did not report or inadequately reported the item. In addition, if the item was not applicable for the study, the item would be excluded from the quality assessment. The scoring process was conducted by two authors (X. L. and W. C.) independently, and any discrepancies were discussed by Y. W., S. W., X. L., and W. C. to achieve consensus.

### 2.6. Risk of Bias Assessment

The six domains of bias in the Cochrane Risk of Bias tool were all included in the assessment: (i) selection bias (random sequence generation, allocation concealment), (ii) performance bias (blinding of participants and personnel), (iii) detection bias (blinding of outcome assessment), (iv) attrition bias (incomplete outcome data), (v) reporting bias (selective reporting), and (vi) other bias. Each domain was given a judgment of “low risk of bias”, “unclear risk of bias”, or “high risk of bias” in line with the criteria in the Cochrane handbook [23] by the two authors (X. L. and W. C.) independently. Any discrepancies were discussed with another author S. W. to achieve consensus.

### 2.7. Synthesis of Results and Statistical Methods

The number of RCTs by nonmarketed/marketed products, formulation, focused functions, publication year, and locations of RCTs was analyzed descriptively to give an overview of the characteristics of the included RCTs.

In the part of quality evaluation, with the above scoring system applying to the quality assessment, each study was given a total score. Due to the different number of applicable items of each study, an average score that ranges from 0 to 1 was finally obtained by dividing the total score by the number of items, with a higher score indicating a higher reporting quality of the study.

To identify sections in which authors could improve the reporting quality, the average CONSORT score on each grouped item (e.g., title and abstract, trial design) was synthesized. Besides, in order to assess the influence of the publication of the CONSORT statement on the reporting quality, the CONSORT scores of RCTs were analyzed by publication years. Pearson's Correlation Coefficient (Pearson's *r*) with 2-tailed significance between the average CONSORT score and the sample size/trial length of RCTs was also performed to explore the correlation between them.

The synthesis of results was all analyzed by grouping RCTs into different *Rhodiola* species to see their respective quality. Excel 16.39 (Microsoft, Redmond, WA, USA), Prism 9.0 (GraphPad Software, San Diego, CA, USA), SPSS 26.0 (IBM, Armonk, NY, USA), and RevMan 5.4 (The Cochrane Collaboration, 2020) were used to perform the analysis.

## 3. Results

### 3.1. Study Selection

**Figure 1:**
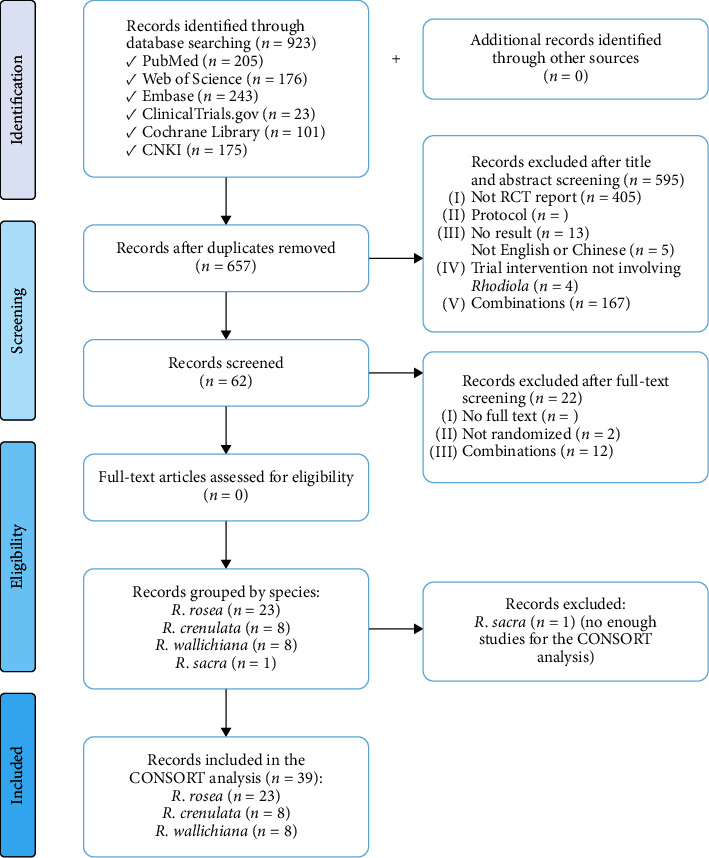
PRISMA flow diagram of study selection.

The study selection process is presented in Figure 1. A total of 923 records were retrieved from the six databases (205 from PubMed, 176 from web of science, 243 from Embase, 23 from ClinicalTrials.gov, 101 from Cochrane Library, and 175 from CNKI), and no additional study was found through other sources. After duplicates were removed, 657 potentially relevant records were assessed for eligibility. A total of 595 of these records were excluded for the following reasons: (i) not RCT reports (403), (ii) protocol (1), (iii) no result presented (13), (iv) not published in English or Chinese (5), (v) not involving *Rhodiola* in the trial intervention (7), and (vi) *Rhodiola* combined with other medicine as therapy intervention (167). The remaining 62 records were subsequently screened with the full text for further exclusion. An additional 22 records were excluded then, including 8 records of which full text was not available, 2 records that were not randomized, and 12 records that involved combination intervention. The remaining 40 records were included after the full-text screening and were grouped into four *Rhodiola* species. As a result, there are 23 records (22 published in English, 1 published in Chinese) of *R*. *rosea*, 8 records of *R*. *crenulata* (2 published in English, 6 published in Chinese), 8 records (all published in Chinese) of *Rhodiola wallichiana* (*R*. *wallichiana*), and 1 record of *R*. *sacra*. Considering that only 1 record of *R*. *sacra* being evaluated is not significant, 1 record of *R*. *sacra* is not included in our quality assessment

### 3.2. Study Characteristics

**Figure 2:**
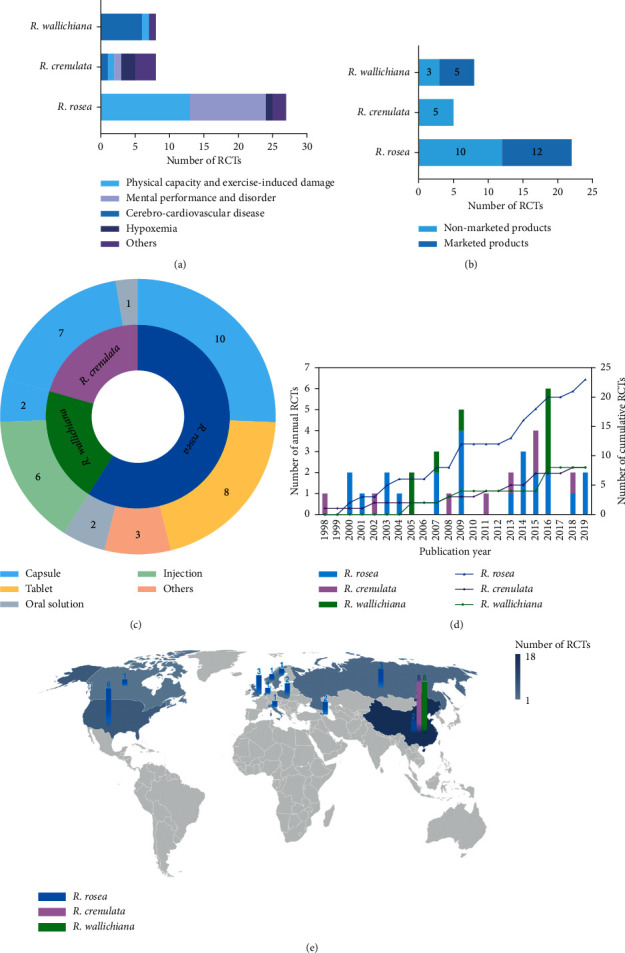
Number of Rhodiola RCTs by focused functions, nonmarketed/marketed products, formulation, publication year, and locations of RCTs. (a) The number of RCTs by focused functions. (b) The number of RCTs by the use of nonmarketed/marketed products as an intervention in trials. (c) The number of RCTs by the formulation of intervention products in trials. (d) The annual/cumulative number of RCTs by the publication year. The blue column represents the annual number of RCTs of *R*. *rosea*. The purple column represents the annual number of RCTs of *R*. *crenulata*. The green column represents the annual number of RCTs of *R*. *wallichiana*. The blue curve represents the cumulative number of RCTs of *R*. *rosea*. The purple curve represents the cumulative number of RCTs of *R*. *crenulata*. The green curve represents the cumulative number of RCTs of *R*. *wallichiana*. (e) The number of RCTs by the locations of the trials.

As illustrated in Figure 2(a), the included 39 RCTs investigated several conditions regarding physical capacity and exercise-induced damage, mental performance and disorder, cerebro-cardiovascular disease, hypoxemia, and others. Among these three *Rhodiola* species, RCTs of *R*. *rosea* mainly focus on physical capacity and exercise-induced damage (*n* = 13), as well as mental performance and disorder (*n* = 11). 4 studies of *R*. *rosea* investigated conditions of both physical capacity and exercise-induced damage and mental performance and disorder, and thus, the total number of *R*. *rosea* studies here in this result is 4 more than the previous 23 studies. As for RCTs of *R*. *wallichiana*, they mainly focus on cerebro-cardiovascular disease (*n* = 6). While for *R*. *crenulata*, except for the 4 conditions mentioned above, RCTs of it also investigated conditions regarding chronic obstructive pulmonary disease (COPD), immunity, and highland alopecia, there is not a focused condition among the above.

The characteristics of the trial interventions of the three *Rhodiola* species were also summarized. In process of collecting data about whether a trial used marketed or nonmarketed products, 1 study of *R*. *rosea* and 3 studies of *R*. *crenulata* were excluded from the analysis due to their missing data regarding the intervention products. As shown in Figure 2(b), for *R*. *rosea* and *R*. *wallichiana*, more than half of the RCTs used marketed *Rhodiola* products (*R*. *rosea* = 12, *R*. *wallichiana* = 5) as the interventions. It is worth noting that the marketed products used in the included RCTs of *R*. *rosea* are all health food, while for those of *R*. *wallichiana*, they are all approved drugs. As for the included five RCTs of *R*. *crenulata*, the intervention products used were all nonmarketed.

Another characteristic of the trial interventions we looked into is the product formulations. As seen in Figure 2(c), the formulations of the intervention products range from the capsule, tablet, oral solution to injection. Among RCTs of *R*. *rosea*, capsule (*n* = 10) and tablet (*n* = 8) are the top two formulations of intervention products. Notably, products of the tablet are only adopted in RCTs of *R*. *rosea*, while products of the capsule are adopted in all three species and are the most commonly used formulation among RCTs of *R*. *crenulata* (*n* = 7). In regard to oral solution, there are only 3 RCTs (*R*. *rosea* = 2, *R*. *crenulata* = 1) that chose the oral solution products as the interventions. In particular, products of injection are only used in RCTs of *R*. *wallichiana* and they account for 75% of the *R*. *wallichiana* RCTs.

A landscape regarding the number of the three *Rhodiola* species RCTs grouped by the publication year is presented in Figure 2(d). Among the included 39 RCTs, an RCT of *R*. *crenulata* published in 1998 is the earliest published, followed by *R*. *rosea* (in 2000) and *R*. *wallichiana* (in 2005). As can be seen from the curve of the number of RCTs cumulated by publication year, the rising trend of RCTs of *R*. *rosea* is the most rapid, while the rising trend of RCTs of the other two species is relatively much slow. The RCTs of *R*. *rosea* were published the most in 2009, with 4 studies. But overall, they were published most intensively from 2013 to 2019, with 11 studies being published in total. Regarding the RCTs of *R*. *wallichiana*, they were published the most in 2016, with 4 studies, while the publication years of RCTs of *R*. *crenulata* were relatively scattered, with the most annual publication of 2 studies in 2015.

A world map in Figure 2(e) presents the trial locations with the number of RCTs. It can be seen that RCTs of both *R*. *crenulata* and *R*. *wallichiana* all took place in China while the locations of RCTs of *R*. *rosea* are across Europe, Asia, and North America, including Russia (*n* = 3), UK (*n* = 3), Poland (*n* = 2), Sweden (*n* = 1), Norway (*n* = 1), Italy (*n* = 1), Netherlands (*n* = 1), Armenia (*n* = 2), China (*n* = 2), USA (*n* = 6), and Canada (*n* = 1). It is obvious that the USA is the country where RCTs of *R*. *rosea* took place most. In addition, locations of RCTs of *R*. *rosea* also clustered in Europe with a number of more than a half.

### 3.3. CONSORT Evaluation

**Figure 3:**
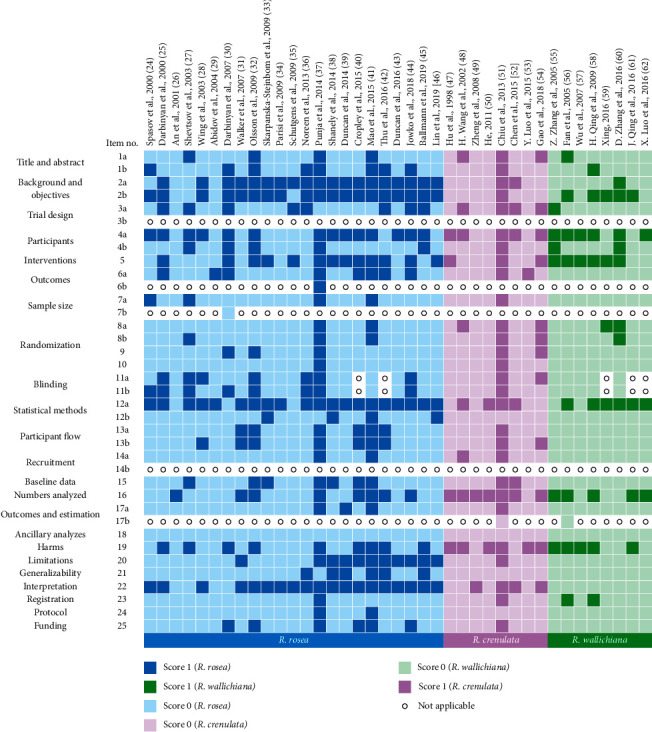
Scoring based on the compliance with the CONSORT statement of each RCT of *R*. *rosea*, *R*. *crenulata*, and *R*. *wallichiana* on subdivided items (Title and abstract: 1a, 1b; Background and objectives: 2a, 2b; Trial design: 3a, 3b; Participants: 4a, 4b; Interventions: 5; Outcomes: 6a, 6b; Sample size: 7a, 7b; Randomization: 8a, 8b (Sequence generation), 9 (Allocation concealment mechanism), 10 (Implementation); Blinding: 11a, 11b; Statistical methods: 12a, 12b; Participant flow: 13a, 13b; Recruitment: 14a, 14b; Baseline data: 15; Numbers analyzed: 16; Outcomes and estimation: 17a, 17b; Ancillary analyses: 18; Harms: 19; Limitations: 20; Generalizability: 21; Interpretation: 22; Registration: 23; Protocol: 24; Funding: 25) [24–62].

In Figure 3, the given scores of each study for each item in accordance with the compliance with the CONSORT statement are presented in the form of a heat map, with the studies sorted by the publication year. Apparently, none of the included studies met all the CONSORT statement criteria. The overall CONSORT compliance of the RCTs of the three *Rhodiola* species is poor as many of the items display a great proportion of light color area which means that the items were inadequately reported. For example, regarding item 1a “Identification as a randomized trial in the title”, only 4 of the 23 RCTs of *R*. *rosea*, 3 RCTs of *R*. *crenulata*, and 1 RCTs of *R*. *wallichiana* reported it. For another item 7a “How sample size was determined”, only 5 RCTs of *R*. *rosea*, 1 RCTs of *R*. *crenulata*, and none of the RCTs of *R*. *wallichiana* reported it. In the case of item 14a “Dates defining the periods of recruitment and follow-up”, only 2 RCTs of *R*. *rosea*, 2 RCTs of *R*. *crenulata*, and none of the RCTs of *R*. *wallichiana* reported it.

**Figure 4:**
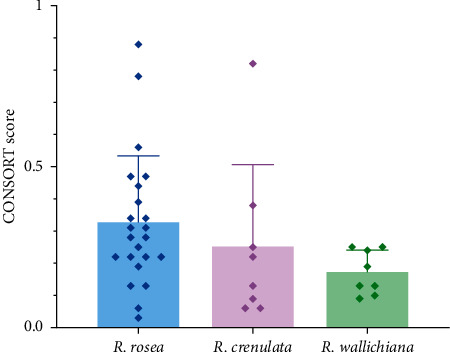
The CONSORT score of each RCT of *R*. *rosea*, *R*. *crenulata*, and *R*. *wallichiana* with the mean score and SD of each species.

The score of each RCT of each species and the average score of each species are presented in Figure 4. The scores of the 39 RCTs range from 0.03 to 0.88, and the overall mean scores for RCTs of *R*. *rosea*, *R*. *crenulata*, and *R*. *wallichiana* are 0.33, 0.25, and 0.17, respectively. In addition, the standard deviation (SD) of scores of *R*. *rosea*, *R*. *crenulata*, and *R*. *wallichiana* is 0.2, 0.25, and 0.07, respectively, which means that the homogeneity among reporting quality of *R*. *rosea* and *R*. *crenulata* is much smaller than that of *R*. *wallichiana*.

**Figure 5:**
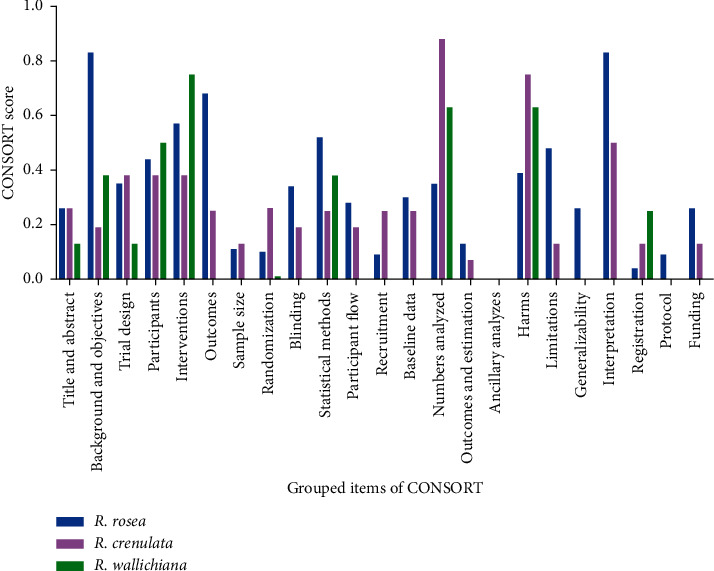
The overall CONSORT score of RCTs of *R*. *rosea*, *R*. *crenulata*, and *R*. *wallichiana* on grouped items of CONSORT.

Although the overall scores of RCTs of the three *Rhodiola* species do not differ from each other much, their scores vary from item to item (Figure 5). Firstly, RCTs of these three species are all in poor compliance with the CONSORT statement regarding the items including title and abstract, sample size, randomization, participant flow, recruitment, outcomes and estimation, ancillary analyses, generalizability, registration, protocol, and funding. Moreover, RCTs of *R*. *crenulata* are also in very poor compliance with the items of background and objective, outcomes, blinding, statistical methods, baseline data, and limitations, while RCTs of *R*. *wallichiana* are also of poor reporting quality regarding the items of background and objective, trial design, outcomes, blinding, baseline data, limitations, and interpretation.

All of the above-highlighted items are with scores below 0.3. On the other hand, there are still several items with relatively good compliance and with scores above 0.7. RCTs of *R*. *rosea* are in good compliance with items including background and objectives and interpretation while for RCTs of *R*. *crenulata*, they are in good compliance with the item of numbers analyzed and harms. As for RCTs of *R*. *wallichiana*, they are in good compliance with the items of interventions.

**Figure 6:**
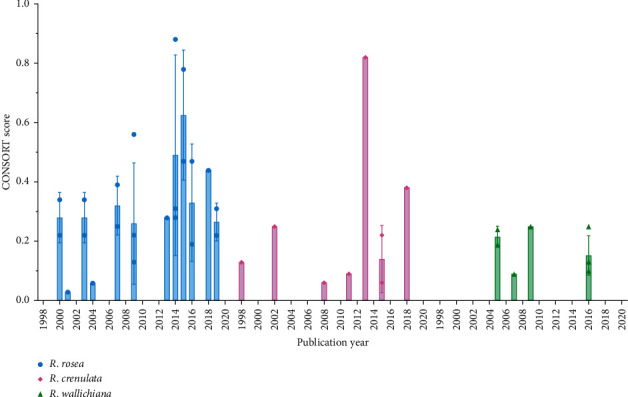
The CONSORT score of each RCT of *R*. *rosea*, *R*. *crenulata*, and *R*. *wallichiana* by publication year (mean ± SD).

In order to explore the impact of publication year on the CONSORT adherence, especially after 2010 when the CONSORT statement was updated, the CONSORT scores of the RCTs of each *Rhodiola* species were classified in years and are presented in Figure 6, as well as the annual average score. As the results of RCTs of *R*. *rosea* show, among the years before 2010, the average scores of 2001 and 2004 are particularly low, which are 0.03 and 0.06, respectively, while the remaining four years (2000, 2003, 2007, and 2009) are around 0.4. As for the years after 2010, the average scores for 2014 and 2015 are 0.49 and 0.63, respectively, making them the two highest-scoring years among all. However, the scores of the remaining years do not improve much compared with those of the years before 2010. For the RCTs of *R*. *crenulata*, the average score of each year before 2010 is below 0.3. Among the years after 2010, the average score of 2013 reaches 0.82, but the scores of the other three years are all low, which are below 0.4. With respect to the RCTs of *R*. *wallichiana*, after 2010, a total of 4 studies were published all in 2016. However, the annual average score only reaches 0.15 which is a bit different from those of the years before 2010. As for the specific items, back to Figure 3, it is obviously that for RCTs of *R*. *rosea*, the compliance with CONSORT on limitations and generalizability has been significantly improved since 2013. To be specific, on the item of limitations, 10 of the 11 RCTs published after 2010 were all rated with score 1 while only 1 RCT published before 2010 was rated with score 1. As for the item of generalizability, 6 of the 11 RCTs published after 2010 were rated with score 1 while none of the RCTs published before 2010 were rated with score 1. However, on other items of RCTs of *R*. *rosea*, as well as the items of RCTs of *R*. *crenulata* and *R*. *wallichiana*, the compliance with CONSORT on them was not improved over time.

**Figure 7:**
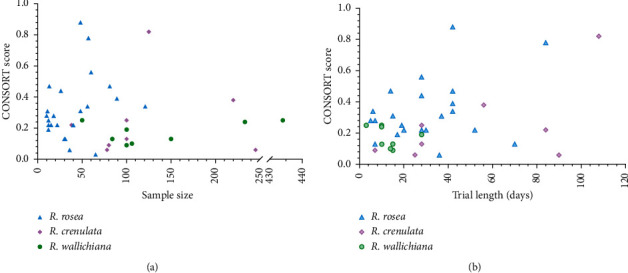
The correlation between the CONSORT score and sample size/trial length. (a) The correlation between the CONSORT score and sample size of RCTs of *R*. *rosea*, *R*. *crenulata*, and *R*. *wallichiana*. (b) The correlation between the CONSORT score and trial length of RCTs of *R*. *rosea*, *R*. *crenulata*, and *R*. *wallichiana*.

In addition to the publication year, the sample size and trial length were also investigated to examine if they have correlations with the CONSORT score (Figures 7(a) and 7(b)). Due to the missed information about the trial length of one RCT of *R*. *rosea*, there were only 22 RCTs of *R*. *rosea* included in Pearson's *r* analysis. Unexpectedly, the results of Pearson's *r* indicated a nonsignificant and weak correlation between the CONSORT score and the sample size of RCTs of *R*. *rosea*, *R*. *crenulata*, or *R*. *wallichiana* (*R*. *rosea*: *r* = 0.265, *p* = 0.222; *R*. *crenulata*: *r* = 0 .088, *p* = 0.836; *R*. *wallichiana*: *r* = 0.49, *p* = 0.218). Likewise, between the CONSORT score and the trial length of RCTs of *R*. *rosea*, *R*. *crenulata*, or *R*. *wallichiana*, a nonsignificant and weak correlation was also found (*R*. *rosea*: *r* = 0.34, *p* = 0.122; *R*. *crenulata*: *r* = 0.6, *p* = 0.116; *R*. *wallichiana*: *r* = −0.333, *p* = 0.42). In Figures 7(a) and 7(b), the correlations between the CONSORT score and the sample size/trial length are graphically presented.

### 3.4. Risk of Bias Assessment

**Figure 8:**
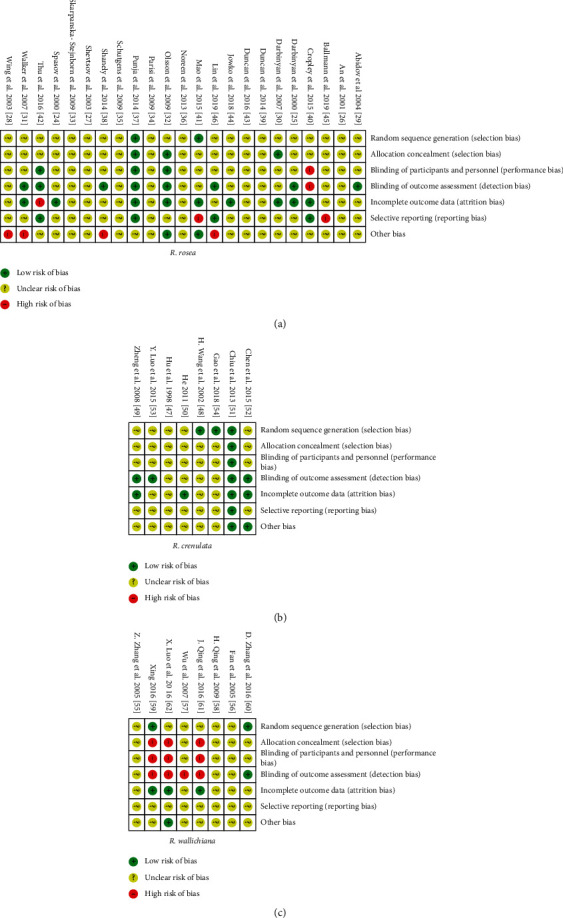
Risk of bias summary for the individual included studies. (a) Risk of bias summary for the individual RCTs of *R*. *rosea*. (b) Risk of bias summary for the individual RCTs of *R*. *crenulata*. (c) Risk of bias summary for the individual RCTs of *R*. *wallichiana*.

**Figure 9:**
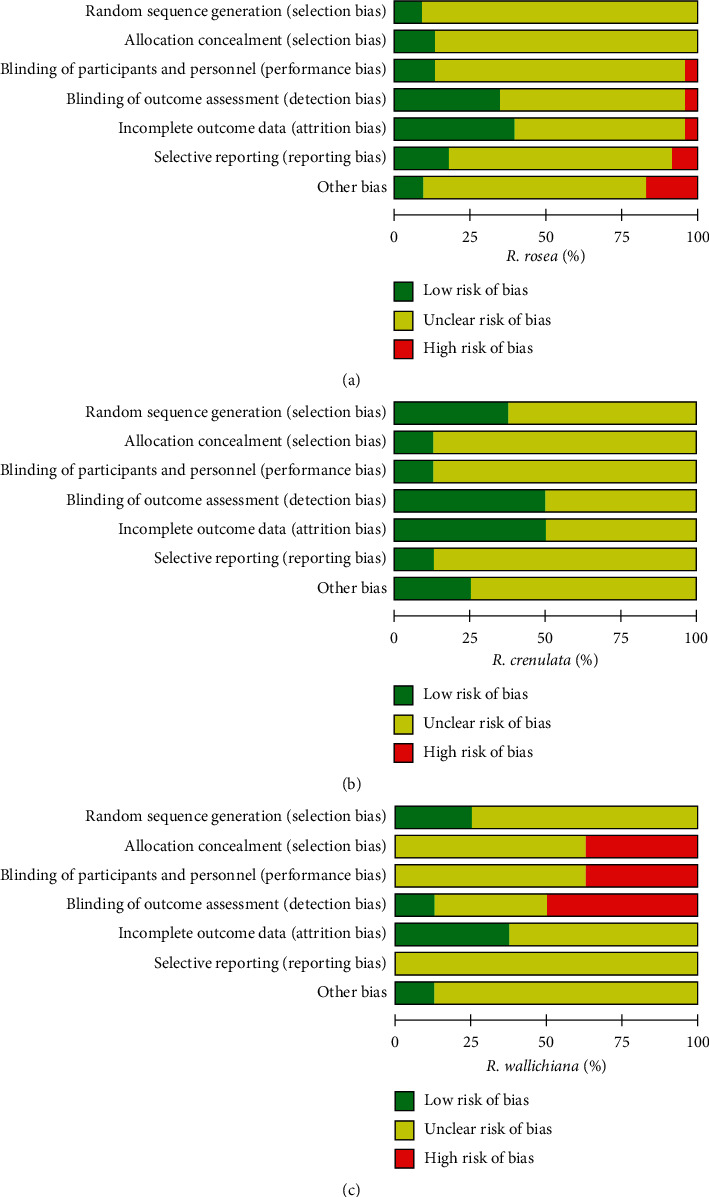
Percentage of risk of bias of the included studies. (a) Percentage of risk of bias of RCTs of *R*. *rosea*. (b) Percentage of risk of bias of RCTs of *R*. *crenulata*. (c) Percentage of risk of bias of RCTs of *R*. *wallichiana*.

As seen from Figure 8 in which the judgment of risk of bias regarding the six domains of the included studies is presented, the majority of the included studies of the three *Rhodiola* species were judged to have an unclear risk of bias in most domains due to the insufficient reporting. Among the studies of *R*. *rosea* and *R*. *wallichiana*, none of them was assessed to have a low risk of bias in all the six domains while one study of *R*. *crenulata* was found to reach a low risk of bias in all the domains. In Figure 9, the judgements of risk of bias were further summarized by percentage. There are studies of *R*. *rosea* which have a high risk of bias in domains of performance bias, detection bias, attrition bias, or reporting bias with percentages not exceeding 9%. For the other risks of bias, approximately 17% of studies of *R*. *rosea* were judged to have high risk of bias due to the industry sponsorship issue [38] or the lack of sample size calculation along with small sample size and nonsignificant results [28, 31, 46]. For both studies of *R*. *crenulata* and *R*. *wallichiana*, no high risk of bias was found during the assessment. For the assessment in studies of *R*. *wallichiana*, the percentages of high risk of bias in selection bias, performance bias, and detection bias are 38%, 38%, and 50%, respectively, which are relatively high compared with the other two species. At last, among the studies of *R*. *crenulata*, no high risk of bias was found.

## 4. Discussion

The current study identified 23 RCTs of *R*. *rosea*, 8 RCTs of *R*. *crenulata*, and 8 RCTs of *R*. *wallichiana* for the evaluation of their reporting quality based on the CONSORT 2010 statement and the assessment of their methodological quality (risk of bias) using the Cochrane Risk of Bias tool. Prior to the CONSORT evaluation and risk of bias assessment, the study characteristics including nonmarketed/marketed products, formulation, focused functions, publication year, and locations of RCTs were investigated to give an overview of the status of current RCTs of *Rhodiola*. The results of the CONSORT evaluation showed that the reporting quality of the included studies was generally poor. Furthermore, most of the included studies were judged to have an unclear risk of bias in most domains due to the limited reporting information in the assessment of Cochrane Risk of Bias.

The distribution of the locations of RCTs of *R*. *rosea*, *R*. *crenulata*, and *R*. *wallichiana* is consistent with the distribution of respective *Rhodiola* resources [9], implying that the *Rhodiola* researchers still focus on utilizing the local resource currently. In western countries, *R*. *rosea* is the most widely used species [63]. Products of *R*. *rosea* have already been on the market, and most of them are sold as food supplements [64]. In China, a wide variety of *Rhodiola* species are used. *R*. *crenulata* is the official species listed in the Chinese Pharmacopoeia (version of 2020), while some other species are also commonly used, such as *R*. *wallichiana*, *R*. *angusta*, and *R*. *sachalinensis* [65, 66]. Specifically, *R*. *wallichiana* is the only species that has been developed to injection for treating stable angina pectoris associated with coronary heart disease [67]. In light of the diverse functions of *Rhodiola*, more and more products have been developed. However, a high-value product, as a drug or functional food, needs support from sufficient clinical trial data. In our study, there are 39 RCTs of *Rhodiola* included, and the number of RCTs of *R*. *rosea* (*n* = 23) is almost three times of the number of RCTs of *R*. *crenulata* (*n* = 8) and *R*. *wallichiana* (*n* = 8), respectively, which means that more RCTs of *R*. *crenulata* and *R*. *wallichiana* are needed.

In addition to sufficient clinical trial data, the quality of clinical trial data is more important for the support of high-value products. However, the overall quality of the current RCTs of *Rhodiola* is poor. Taking the item “Title and abstract” as an example, it seems very easy to be adherent to; however, the CONSORT scores of the RCTs of the three species are all below 0.3 which means poor compliance with this item. Only a total of 8 RCTs were identified as the randomized trials in the title. For the 26 RCTs which did not fully report item 1b “Structured summary of trial design, methods, results, and conclusions”, most of them lacked the information about the trial design. Actually, the title and abstract are the important parts of a study. In a conventional literature screening method when conducting a systematic review for RCTs, a reviewer usually decides whether to include a study for further screening based on the examination of the title and abstract [68]. Those studies that have identified randomization in the title and have structured abstracts with sufficient information will help reviewers quickly screen the literature in the initial screening. Otherwise, there is a possibility that the studies will be excluded by the reviewers. Furthermore, an abstract with insufficient information or inappropriate presentation might sometimes mislead those decision-makers who cannot get access to the full-text reports and make a wrong healthcare decision [69]. Therefore, the title and abstract should be paid sufficient attention to by the researchers when they write the trial report.

In the method section, sample size calculation is another item with poor CONSORT compliance in the present study. In total, only 6 (15%) articles (5 RCTs of *R*. *rosea*, 1 RCT of *R*. *crenulata*, and 0 RCTs of *R*. *wallichiana*) reported how the sample size was determined. In line with our study, several other studies also found that the calculation of sample size was seldom reported [13, 70, 71]. A study also pointed out that researchers, reviewers, and editors do not attach much importance to the sample size calculation [72]. However, the sample size calculation is actually of importance. In our study, three RCTs reporting nonsignificant results were judged to have a high risk of bias just because they recruited a small sample of participants and did not report the sample size calculation. A too small sample size often fails to detect statistically significant relation or difference, which is also well known as Type II error [73]. Consequently, a null trial that uses a small sample size and does not specify the sample size calculation will raise the reviewers' concern about the validity of its results. However, an overlarge sample size could magnify the detection of differences, highlighting statistical differences which are not clinically relevant. Besides, it would also cause waste of budget and could involve ethical problems. Hence, how the sample size was calculated should be reported so that the reviewers can examine whether the sample size of the study is sufficient and appropriate.

Randomization and blinding are crucial parts of the methodology in an RCT report. Detailed information of randomization and blinding being reported can reduce the bias and thus improve the validity of the study [74]. However, the RCTs of *Rhodiola* seem to have a low quality of these two important items. A study conducted by Ishaque et al. [20] evaluated the safety and efficacy of *R*. *rosea* for mental and physical fatigue by systematically reviewing the clinical trials of *R*. *rosea*. The results showed that the majority of the clinical trials unclearly reported the method of randomization and allocation concealment, and almost half of the included studies had an unclear or high risk of bias of blinding. Likewise, a meta-analysis of *R*. *wallichiana* preparation in the treatment for unstable angina pectoris also indicated the insufficient reports of the randomization method among the included RCTs [65]. Consistent with the results of the above two studies, in this study, the compliance with randomization and blinding of the included RCTs is also poor. The item randomization in the CONSORT statement is composed of 4 items with respect to sequence generation, allocation concealment mechanism, and implementation. Among all the RCTs included, only 2 (1 RCT of *R*. *rosea* and 1 RCT of *R*. *crenulata*) fully complied with all 4 items of randomization. 29 RCTs even did not report any of the 4 items at all. As for blinding, likewise, the majority of the included RCTs of the three *Rhodiola* species did not adequately report the blinding information, and the risk of bias regarding blinding of participants and personnel and blinding of outcome assessment is even high in 38% and 50% of RCTs of *R*. *wallichiana*, respectively. The high risk of bias was attributed to the incomplete blinding or lack of blinding. To improve the validity of RCTs of *Rhodiola*, researchers should give enough attention to the improvement of randomization and blinding.

In clinical trials, the report of adverse events is essential since safety is the basic requirement of a drug. Nevertheless, in the current study, nearly half of the included RCTs (15 RCTs of *R*. *rosea*, 1 RCT of *R*. *crenulata*, and 3 RCTs of *R*. *wallichiana*) did not mention if there were adverse events. As can be seen from the above results, the compliance with harms of RCTs of *R*. *rosea* is poorer than that of the RCTs of the other two species. This might attribute to the fact that most of the RCTs of *R*. *rosea* examined the effect on improving mental or physical function, rather than treating a certain disease. Researchers may, therefore, not take the adverse events seriously enough. In contrast to the RCTs of *R*. *rosea*, RCTs of *R*. *crenulata* and *R*. *wallichiana* focused more on treating diseases, such as cerebro-cardiovascular disease and COPD. Therefore, researchers were more cautious in observing and reporting any adverse events, which resulted in a higher proportion of reporting harms. However, no matter whether the RCT investigates a disease or not, the reporting of adverse events should be paid enough attention to, especially for herbal drugs, since there are many kinds of ingredients in herb and some of them are even not acquainted by scientists. Taking *Rhodiola* as an example, there are more than 100 ingredients reported [9]. This is different from western medicine in which ingredients are relatively few and the effects are easier to be predicted. In addition, among the RCTs of *R*. *wallichiana*, 6 used *R*. *wallichiana* injections as interventions. Disappointingly, 3 of them did not mention any detail of the harms. The safety of herbal injections has become a public concern since the Yuxingcao injection caused a series of severe adverse events and was subsequently suspended in 2006 [75]. Herbal injections are considered to have a higher risk of adverse events than any other formulation of herbal drugs and conventional injections [76]. In view of the above, adverse effects should be paid great attention to when conducting relevant clinical trials.

The overall qualities of RCTs of the three *Rhodiola* species range from 0.17 to 0.32, which means that they do not differ from each other too much. Nevertheless, on some items such as background and objectives, blinding, outcomes, and interpretation, differences among their qualities are significant. Overall, CONSORT grouped items of RCTs of *R*. *rosea* and *R*. *crenulata* were nearly all reported, indicating that RCT reports of *R*. *rosea* and *R*. *crenulata* are with a relatively complete structure based on the CONSORT. As for RCT reports of *R*. *wallichiana*, more than half of the grouped items were not reported at all. Authors should give adequate attention to each item on the CONSORT checklist. An RCT report with a complete structure of the CONSORT and adequate reporting of each item can provide the reviewers with sufficient information to evaluate the effectiveness of the RCT.

The reporting quality of RCTs is believed to be affected by some factors, such as sample size. A large and appropriate sample size is considered to be associated with better quality of RCT reporting [77]. In a previous study conducted by Kodounis et al. [14], a significant association was found between sample size and the quality of reporting in a univariate analysis. It is not hard to explain and understand this association. As the sample size increases, the expenditure and manpower invested in RCTs will also increase, which results in an aspiration of researchers to produce a good quality RCT report. Therefore, from this perspective, the increase in sample size is believed to improve the quality of reporting. With the same logic, we believe that the increase in trial length can also improve the quality of reporting. Thus, we investigated the correlation between the CONSORT score and the sample size, as well as the trial length, respectively. However, the results showed weak correlations, either for sample size or for trial length. Compared with Kodounis's study [14], our limited sample size of the included RCTs may be responsible for the result of the weak correlation. Additionally, the publication year is possibly a confounding factor of this negative result. The quality of reporting is supposed to improve over time, especially after 2010 when the CONSORT statement was updated for a more complete version. Among the included RCTs in our study, however, the CONSORT scores of most RCTs published after 2010 are actually similar to those of the RCTs published before 2010. Several RCTs published after 2010 are even in poorer compliance compared with some RCTs published before 2010 based on the CONSORT score. But three RCTs with high scores which are above 0.8 were not published after 2010. In addition, on specific items, the compliance with only two items of RCTs of *R*. *rosea*, such as limitations and generalizability, has been improved significantly after the CONSORT statement was updated in 2010. Obviously, the CONSORT 2010 statement has a limited influence on the quality of reporting among RCTs of *Rhodiola*.

To improve compliance with CONSORT, researchers should be trained with CONSORT before they conduct the clinical trial and take the CONSORT seriously when they write the report. In addition to the effort the researchers should make, journals can also make contributions. A previous study has revealed that the adoption of CONSORT by journals is related to the improvement of the quality of RCT reports [78]. Specifically, the poor CONSORT compliance is sometimes attributed to the restriction of journal format and word count, such as the part of title and abstract [79]. To resolve this problem, in addition to condensing the language on the basis of keeping the complete information by authors, journals are suggested to adjust the format and word count in response to the CONSORT, and the submitted manuscript should have high CONSORT compliance.

Only when the transparency of RCT reports is guaranteed can the risk of bias of the report be assessed properly. In our study, due to the inadequate reporting of the included RCTs, the risk of bias of most of them in most domains was assessed to be unclear, indicating the unclear methodological quality. Meanwhile, some RCTs of *R*. *rosea* and *R*. *wallichiana* were found to have a high risk of bias regarding the allocation concealment, blinding of participants and personnel, blinding of outcome assessment, incomplete outcome data, selective reporting, funding, and sample size calculation, which lower the validity of those studies. For the RCTs of *R*. *crenulata*, although no high risk of bias was found among them, the validity of their results should be taken with a grain of salt due to a large proportion of the unclear risk of bias. Overall, the results of the included RCTs of *Rhodiola* which were evaluated to have an unclear or high risk of bias should be interpreted with caution.

In this study, there are also some limitations. Firstly, we planned to cover the RCTs of all the *Rhodiola* species, but eventually, there are only three species included in the study, and one species was excluded due to limited trials. The RCTs of other *Rhodiola* species may be missed due to the limited included publication language. In journals of other publication languages, there may be more eligible RCTs of the current included three species. Secondly, only peer-reviewed and full-text journal articles were included. Those reports such as conference abstracts are usually not full-text available while the CONSORT statement and Cochrane Risk of Bias are only suitable for the quality evaluation for full-text trial reports. In addition, the articles that have not been peer-viewed will raise certain concerns about the validity. In view of these concerns, we only included peer-reviewed and full-text journal articles, and the quality of those articles without peer review and full text remains unknown. Thirdly, the sample size in our study is relatively small, especially for RCTs of *R*. *crenulata* and *R*. *wallichiana*. In the future, when *Rhodiola* is investigated further, more RCTs would be included in the quality assessment for further investigation. Finally, we only included RCTs of *Rhodiola* single treatment. The quality of RCTs with *Rhodiola* combination treatment remains unknown. Therefore, the result of this study only represents the quality of RCTs with *Rhodiola* single treatment published in English and Chinese.

## 5. Conclusion

According to the reporting quality evaluation in this study, there is inadequate reporting among the included RCTs of *R*. *rosea*, *R*. *crenulata*, and *R*. *wallichiana*. Furthermore, in the assessment of the risk of bias, most of the included studies were found to have an unclear risk of bias, which raises the concern about their validity. Therefore, *Rhodiola* researchers should use these clinical pieces of evidence with caution, and more RCTs with high reporting quality and good methodological quality are needed. In order to achieve the high quality of RCTs of *Rhodiola*, researchers are suggested to rigorously comply with the CONSORT statement when designing the trial and writing the report. We believed that with the improvement of the quality of RCTs, the development of *Rhodiola* products will attract more attention and be of higher value.

## Data Availability

All data generated or analyzed during this study are included in this published article.
